# Clinical features and treatment efficacy for IgG4-related thyroiditis

**DOI:** 10.1186/s13023-021-01942-x

**Published:** 2021-07-21

**Authors:** Xinxin Han, Panpan Zhang, Jieqiong Li, Zheng Liu, Hui Lu, Xuan Luo, Boju Pan, Xiaolan Lian, Xuejun Zeng, Wen Zhang, Xiaofeng Zeng

**Affiliations:** 1 Department of General Practice, Peking Union Medical College Hospital, Chinese Academy of Medical Science and Peking Union Medical College, State Key Laboratory of Complex Severe and Rare Diseases, Beijing, People’s Republic of China; 2 Department of Rheumatology, Peking Union Medical College Hospital, Chinese Academy of Medical Science and Peking Union Medical College, Key Laboratory of Rheumatology and Clinical Immunology, State Key Laboratory of Complex Severe and Rare Diseases, Ministry of Education and National Clinical Research Center for Dermatologic and Immunologic Diseases (NCRC-DID), No.1 Shuai Fu Yuan, Dong Cheng District, Beijing, 100730 People’s Republic of China; 3 Department of Pathology, Peking Union Medical College Hospital, Chinese Academy of Medical Science and Peking Union Medical College, State Key Laboratory of Complex Severe and Rare Diseases, Beijing, People’s Republic of China; 4 Department of Endocrine, Peking Union Medical College Hospital, Chinese Academy of Medical Science and Peking Union Medical College, State Key Laboratory of Complex Severe and Rare Diseases, Beijing, People’s Republic of China

## Abstract

**Purpose:**

This study aimed to clarify the clinical features of and evaluate the treatment efficacy for IgG4-related thyroiditis.

**Methods:**

Fourteen IgG4-related thyroiditis patients and 42 randomly matched IgG4-related disease (IgG4-RD) patients without thyroiditis in a prospective cohort at the Peking Union Medical College Hospital (PUMCH) were enrolled from 2011 to 2019. Patient demographics, clinical characteristics, laboratory parameters and treatment efficacy were analysed.

**Results:**

The prevalence of IgG4-related thyroiditis in our cohort was 2.0%. The average patient age was 42.8 ± 14.9 years, and the male: female ratio was 1:1. Goiter (14, 100.0%), hard thyroid (14, 100.0%) and neck compression (5, 35.7%) were the most prevalent onset symptoms observed. IgG4-related thyroiditis was characterized by asymmetric diffuse thyroid enlargement on ultrasound. Thirteen (92.9%) patients had hypothyroidism, and all patients had significantly elevated circulating thyroid antibodies. Compared with patients without thyroiditis, patients with IgG4-related thyroiditis had less submandibular gland involvement and lacrimal gland involvement and lower serum IgG4 and T-IgE levels (*P* = 0.019, *P* = 0.022, *P* = 0.004, and *P* = 0.006, respectively) and more single-organ involvement (*P* = 0.011). After treatment, the symptoms were relieved, while the size of the thyroid gland did not change significantly, and levothyroxine as a supplemental therapy was still needed.

**Conclusions:**

IgG4-related thyroiditis is a distinct subtype of IgG4-RD characterized by positive circulating thyroid antibodies and a high rate of hypothyroidism. Although compression symptoms could be relieved with treatment, the thyroid size did not change significantly, and the damage to thyroid function was often irreversible.

**Supplementary Information:**

The online version contains supplementary material available at 10.1186/s13023-021-01942-x.

## Introduction

IgG4-related disease (IgG4-RD) is an immune-mediated fibroinflammatory disorder [1, 2]. IgG4-RD is characterized by tumefactive swelling of affected organs, elevated serum IgG4, dense lymphocyte infiltration and IgG4-positive plasma cells in tissues [3, 4]. It is a highly heterogeneous disease that can affect nearly any organ and often presents with multi-organ involvement [5, 6]. Accumulating evidence has revealed that a small population of patients with thyroiditis have elevated serum IgG4 and tissue infiltration of IgG4-positive plasma cells [7–9]. The thyroid gland is recognized as an entity of IgG4-RD, although the spectrum of IgG4-related thyroiditis remains to be defined [10].

As one of the less commonly involved organs of IgG4-RD, IgG4-related thyroiditis mainly includes Hashimoto’s thyroiditis (HT) and Riedel’s thyroiditis (RT) [7, 11]. Awareness of this distinct entity may help clinicians guide treatment strategies. IgG4-related HT was first reported by Li et al. [12]. Recognized as a subtype of IgG4-RD, IgG4-related HT is associated with a higher percentage of subclinical hypothyroidism than HT without IgG4 infiltration [11]. Based on pathological and serological findings, Dahlgren et al. and Pusztaszeri et al. reported that RT is a rare form of IgG4-RD involving the thyroid [13, 14]. The management of IgG4-related thyroiditis could be challenging due to the lack of available information about this disease [15].

Currently, the clinical manifestations and pathological features of IgG4-related thyroiditis have been studied [16]. However, the long-term treatment efficacy and similarities and differences in IgG4-RD with/without thyroid involvement are unclear. Therefore, to further understand the characteristics and treatment response of IgG4-related thyroiditis, we summarized its clinical manifestations and evaluated treatment efficacy. In addition, the similarities and differences between IgG4-RD patients with/without thyroiditis were compared.

## Methods

### Patient enrolment

In our prospective cohort study of IgG4-RD carried out in the Peking Union Medical College Hospital (registered at Clinical Trials.gov ID: NCT01670695), 710 IgG4-related disease patients fulfilling the 2011 comprehensive diagnostic criteria were enrolled from January 2011 to November 2019. The diagnosis of IgG4-RD was based on the following criteria: (1) a clinical examination showing characteristic diffuse/localized swelling or masses within single or multiple organs; (2) an elevated serum IgG4 concentration (> 135 mg/dL); and (3) a histopathologic examination showing (a) marked lymphocytic and plasma cell infiltration and fibrosis or (b) infiltration of IgG4+ plasma cells (a ratio of IgG4+ /IgG+ cells > 40% and > 10 IgG4+ plasma cells per high-power field) [9]. Affected organs and treatment efficacy were determined by clinical symptoms, physical examinations, laboratory results, histological pathology and imaging, including ultrasound, computed tomography (CT), magnetic resonance imaging (MRI) or positron emission tomography/computed tomography (PET/CT).

A patient was diagnosed with IgG4-related thyroiditis if he/she fulfilled the diagnostic criteria and had a hard thyroid enlarged in size confirmed by a physical examination and imaging. A hard thyroid was defined as hardness of the thyroid between the forehead and the tip of the nose on the physical examination. Neck compression was based on the patient's chief complaint and the tracheal compression sign by an imaging examination (X-ray or CT scan) due to increased thyroid volume.

According to Zhang and Stone’s review, IgG4-RD consists of two distinct and overlapping subsets: the proliferative type and the fibrotic type [4]. In our cohort, 710 untreated IgG4-RD patients were enrolled: 540 with the proliferative type and 170 with the fibrotic type (IgG4-related fibrosing mediastinitis, thyroiditis, retroperitoneum fibrosis, aortitis, pachymeningitis, sclerosing mesenteritis) or a mixture of both types. To remove confounding factors, patients in the control group were selected from those with proliferative type IgG4-RD (540 patients). In our study, 14 patients with IgG4-related thyroiditis were enrolled, and 42 were selected as controls. A random number table was used to select the 42 patients from the 540 patients.

This study was conducted in compliance with the Declaration of Helsinki and was approved by the Ethics Committee of Peking Union Medical College Hospital (No. S-442). All patients signed written informed consent forms.

### Assessment of thyroid volume by three-dimensional ultrasound

The thyroid lobes were scanned separately, and a transverse scan of the entire thyroid lobe and half of the isthmus was performed through a single sweep from the superior border to the inferior border. The longitudinal, transverse and coronal boundaries of the thyroid lobes were measured, and the volume was calculated with built-in software [17].

### Clinical data and laboratory parameters

Patient data, including age, sex, disease duration, history of allergies, treatment strategy, onset of symptoms, organs affected, and follow-up time, were collected. The IgG4-related disease Responder Index (IgG4-RD RI, 2018 version) and Physician Global Assessment (PGA) at baseline and each follow-up were evaluated [18]. Laboratory parameters included thyroid function; serum thyroglobulin autoantibodies (Tg-Abs) and thyroid peroxidase antibodies (TPO-Abs); routine blood analysis; liver function; kidney function; serum IgG, IgA, and IgM; serum IgG subclasses; total serum IgE (T-IgE); rheumatoid factor (RF), C3 and C4; erythrocyte sedimentation rate (ESR); and hypersensitive C-reactive protein (hsCRP) tests.

### Assessment of treatment efficacy

Treatment response was assessed by evaluating the changes in symptoms, size and hardness of the thyroid gland, thyroid function, and IgG4-RD RI scores [19]. Clinical relapse was defined as the reappearance of clinical symptoms or worsened imaging findings with or without elevated serum IgG4 levels [19, 20].

### Statistical analyses

Statistical analyses were performed using IBM SPSS Statistics version 24.0 software (IBM, Armonk, NY, USA). Data are reported as the mean ± standard deviation or the median and range (interquartile range or min–max). Normally distributed data between two groups were analysed using independent samples t-tests or paired samples t-tests, and one-way analysis of variance was used to compare groups. Categorical data were analysed using the chi-square test or Fisher’s exact tests, while non-normally distributed data were analysed using the rank-sum test. A two-tailed *P*-value ≤ 0.05 was considered statistically significant.

## Results

### Demographic characteristics of IgG4-related thyroiditis patients

Among the 710 IgG4-RD patients, 14 (7 men and 7 women) were diagnosed with IgG4-related thyroiditis. The demographic features of patients with IgG4-related thyroiditis are shown in Table 1. The mean patient age was 42.8 ± 14.9 years, with a male/female ratio of 1:1. The median follow-up time was 25.5 (12.8–39.8) months. For patients with IgG4-related thyroiditis, the mean IgG4-RD RI and PGA were 4.7 ± 3.5 and 4.0 ± 2.4 at baseline, respectively. Moreover, 5 (35.7%) IgG4-related thyroiditis patients had a history of allergies. The mean number of organs affected was 2.1 ± 1.4. Of the patients with IgG4-related thyroiditis, 7 (50.0%) had thyroiditis alone, while 7 (50.0%) had multiple organ involvement.

**Table 1:** Demographic features of patients with/without IgG4 related thyroiditis

Demographic features	IgG4-related thyroiditis (n = 14)	IgG4-RD without thyroiditis (n = 42)	*P* value
Age (years)	42.8 ± 14.9	54.3 ± 16.1	0.022*
Disease duration(month), M (Q1–Q3)	49 (6–99)	30 (6–36)	0.109
Male/female	1:1	1.8:1	0.363
Baseline IgG4-RD RI	4.7 ± 3.5	8.2 ± 4.2	< 0.001*
Baseline PGA	4.0 ± 2.4	6.7 ± 2.4	< 0.001*
Number of organs affected (median, min–max)	2 (1–6)	3 (1–7)	0.010*
History of allergy (n, %)	5 (35.7)	21 (50.0)	0.537
Number of single organ involvement (n, %)	7 (50.0)	6 (14.3)	0.011*

IgG4-RD RI represented IgG4-RD responder index; PGA: physician's global assessment

*Represented statistical significance

**Fig. 1:**
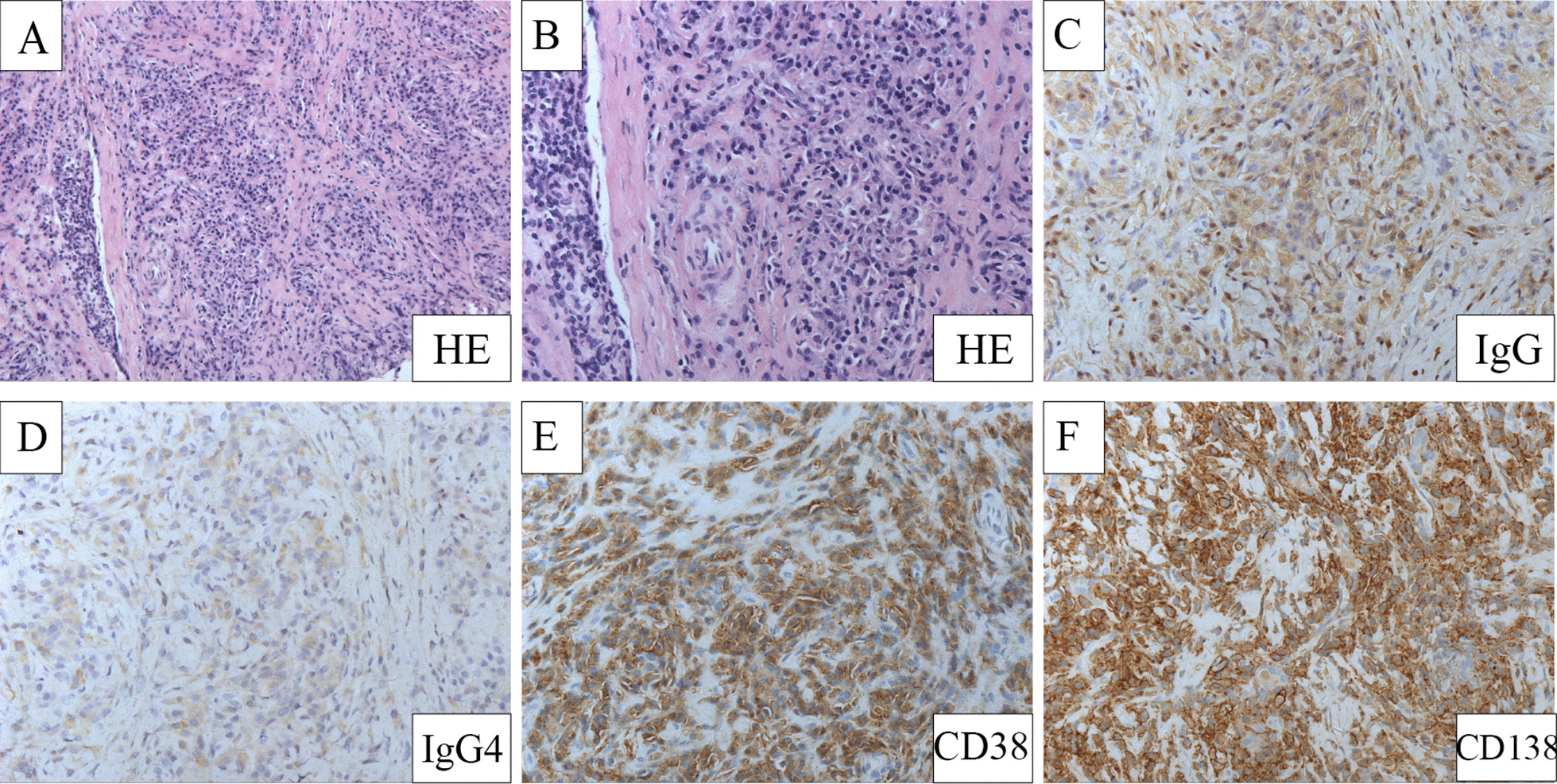
Characteristic pathological features of 1 IgG4-related thyroiditis patient. **A** Hematoxylin and eosin staining showed dense lymphoplasmacytic infiltration and fibrosis (200×). **B** Hematoxylin and eosin staining (100×). **C** IgG staining, dense infiltration of IgG+ plasma cells (200×). **D** IgG4+ staining, massive IgG4+ plasma cells infiltration (200×). **E** CD38 staining (200×). **F** CD138 staining (200×)

Of the 14 patients with thyroiditis, 6 (42.8%) were diagnosed with definite IgG4-RD, and 8 (57.1%) were diagnosed with possible IgG4-RD. Pathological diagnosis was performed in 8 patients; among them, 7 underwent thyroid biopsies, and 1 underwent a liver biopsy. The representative pathological features of the thyroid of 1 patient are shown in Fig. 1. Histological findings showed dense lymphoplasmacytic infiltration along with storiform fibrosis (Fig. 1a, b). Immunohistochemical staining (IHC) showed CD38+, CD138+, IgG+, and IgG4+ plasma cell infiltration (Fig. 1c–f). The ratio of IgG4+ plasma cells/IgG+ plasma cells was > 40%, and there were > 10 IgG4+ plasma cells/HPF.

### Symptoms of IgG4-related thyroiditis

Symptoms of IgG4-related thyroiditis at baseline are shown in Table 2. Hard thyroid (14, 100.0%), goiter (14, 100.0%) and neck compression (5, 35.7%) were the most prevalent local symptoms observed. Lower limb edema (2, 14.3%), chills (1, 7.1%), dyspnea (1, 7.1%) and fatigue (1, 7.1%), all related to thyroid function, were the most prevalent systemic symptoms observed. Other symptoms at baseline included lymph node swelling (2, 14.3%), lacrimal gland enlargement (1, 7.1%), abdominal pain (1, 7.1%), arthralgia (1, 7.1%), cough (1, 7.1%) and nausea and vomiting (1, 7.1%).

**Table 2:** Baseline symptoms and organs affected of IgG4-RD patients with/without thyroiditis

Symptoms and organs affected at baseline	IgG4-related thyroiditis (n = 14)	IgG4-RD without thyroiditis (n = 42)	*P* value
*Symptoms at baseline (n, %)*			
Goiter	14 (100.0)	0 (0)	< 0.001*
Hardened thyroid	14 (100.0)	0 (0)	< 0.001*
Neck compression	5 (35.7)	0 (0)	0.001*
Lower limb edema	2 (14.3)	2 (4.8)	0.258
Lymph node swelling	2 (14.3)	9 (21.4)	0.711
Lacrimal gland enlargement	1 (7.1)	17 (40.5)	0.023*
Abdominal pain	1 (7.1)	10 (23.8)	0.258
Arthralgia	1 (7.1)	3 (7.1)	1.000
Cough	1 (7.1)	7 (16.7)	0.664
Nausea and vomiting	1 (7.1)	5 (11.9)	1.000
Chills	1 (7.1)	0 (0)	0.250
Dyspnea	1 (7.1)	0 (0)	0.250
Fatigue	1 (7.1)	0 (0)	0.250
Submandibular gland enlargement	0 (0)	14 (33.3)	0.012*
Jaundice	0 (0)	5 (11.9)	0.316
Parotid gland enlargement	0 (0)	3 (7.1)	0.565
Nasal congestion	0 (0)	7 (16.7)	0.174
*Organs affected (n, %)*			
Lymph node	4 (28.6)	18 (42.9)	0.267
Pancreas	1 (7.1)	19 (45.2)	0.011*
Submandibular gland	1 (7.1)	17 (40.5)	0.019*
Lacrimal gland	1 (7.1)	16 (38.1)	0.022*
Lung	1 (7.1)	16 (38.1)	0.021*
Kidney	1 (7.1)	6 (14.2)	0.657
Bile duct	0 (0)	11 (26.2)	0.025*
Parotid gland	0 (0)	5 (11.9)	0.307
Nasal sinus	2 (14.3)	11 (26.2)	0.480
Prostate	0 (0)	6 (22.2)	0.306
Pituitary	2 (14.3)	1 (2.4)	0.151
Periaortitis/periarteritis	1 (7.1)	2 (4.8)	1.000
Pachymeningitis	1 (7.1)	0 (0)	0.250
Liver	1 (7.1)	1 (2.4)	0.441

^*^Represented statistical significance

### Organs affected at baseline in patients with IgG4-related thyroiditis

In addition to the thyroid, other affected organs were as follows: lymph node (28.5%), nasal sinus (14.3%), pituitary (14.3%), pancreas (7.1%), submandibular gland (7.1%), lacrimal gland (7.1%), lung (7.1%), kidney (7.1%) and periaortitis/periarteritis (7.1%) (Table 2).

### Laboratory parameters of IgG4-related thyroiditis

The majority of patients (13, 92.8%) had hypothyroidism, and only 1 (7.1%) patient had normal thyroid function. All patients had significantly elevated levels of thyroid autoantibodies, including Tg-Abs (3172 ± 1516 IU/ml) and TPO-Abs (475 ± 159 IU/ml).

Of the patients with IgG4-related thyroiditis, the mean ESR and hsCRP levels were 27 (14–36) mm/h and 2.06 (0.33–3.99) mg/L, respectively. The mean serum IgG, IgG4 and T-IgE levels were 23.56 ± 8.14 g/L, 4750 (2503–8505) mg/L and 100.3 (16.4–175.0) KU/L, respectively. There was no significant difference between male and female patients in laboratory parameters.

### Imaging findings of IgG4-related thyroiditis

Thirteen (92.8%) patients with IgG4-related thyroiditis underwent thyroid ultrasound scans. The results revealed asymmetric diffuse enlargement of thyroids, decreased echogenicity and uneven echogenicity in all patients. The average volumes of the thyroid glands were 71.62 ± 42.73 cm^3^ (right lobe) and 46.95 ± 26.66 cm^3^ (left lobe), with maximum volumes of 143.89 cm^3^ (right lobe) and 80.51 cm^3^ (left lobe) in one patient. Thyroid cystic nodules were found in 5 patients (36.3%). Four patients (30.8%) had cervical lymph node swelling. Colour Doppler flow imaging (CDFI) demonstrated increased blood flow signals in the thyroid glands of 6 (46.2%) patients.

**Fig. 2:**
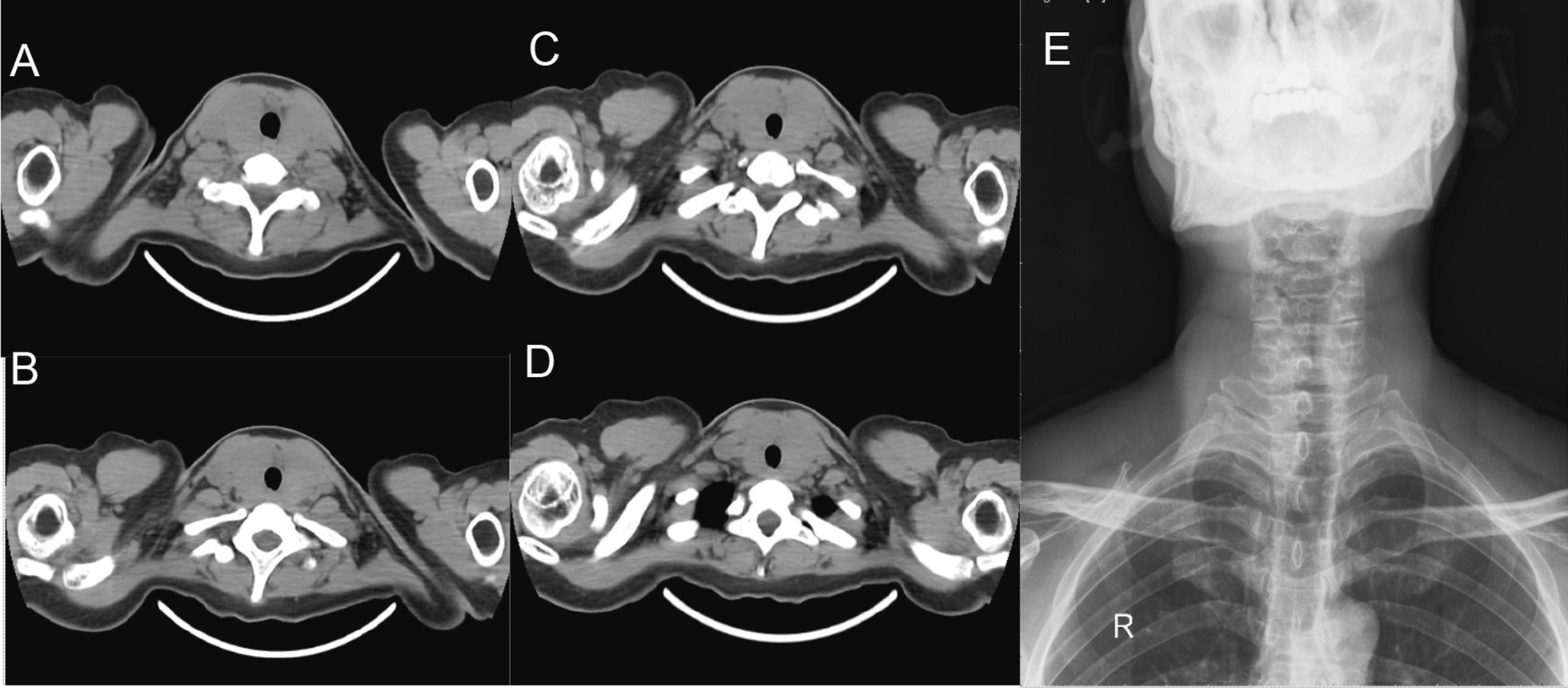
Characteristic imaging findings of IgG4-related thyroiditis. **A**–**D** represented increased thyroid volume with decreased density in CT images of 4 IgG4-related thyroiditis patients. **E** Showed tracheal compression due to increased thyroid volume

Characteristic imaging findings of IgG4-related thyroiditis are shown in Fig. 2. Four (28.6%) patients with IgG4-related thyroiditis underwent CT scans; all patients showed increased thyroid volume with decreased density, and one patient had tracheal compression. One (7.1%) patient who underwent a PET-CT scan showed enlargement of the thyroid gland and increased uptake of 18F-fluorodeoxygluxose (FDG).

### Comparison of IgG4-RD patients with/without thyroiditis

Compared to IgG4-RD patients without thyroiditis, patients with thyroiditis were younger at disease onset and had fewer organs involved, a lower IgG4-RD RI, a lower PGA, and more single-organ involvement (*P* = 0.022, *P* = 0.010, *P* < 0.001, *P* < 0.001, and *P* = 0.011, respectively; Table 1). The male/female ratio, disease duration, and percentage of patients with a history of allergies were comparable between IgG4-RD patients with and without thyroiditis.

With regard to symptoms at disease onset, fewer patients with IgG4-related thyroiditis had submandibular gland and lacrimal gland enlargement than those without (*P* = 0.012, *P* = 0.023, respectively; Table 2). Consistent with these results, fewer patients with IgG4-related thyroiditis had submandibular gland, lacrimal gland, lung, bile duct and pancreas involvement than those without (*P* = 0.019, *P* = 0.022, *P* = 0.021, *P* = 0.025 and *P* = 0.011, respectively; Table 2).

In terms of laboratory findings, compared with those without thyroiditis, patients with IgG4-related thyroiditis had higher levels of platelets (PLTs) (*P* = 0.013), lower levels of serum IgG4 and IgG3, and lower levels of T-IgE (*P* = 0.004, *P* = 0.050 and *P* = 0.006, respectively) (Table 3).

**Table 3:** Laboratory parameters of IgG4-RD with/without thyroiditis

Parameters	IgG4-related thyroiditis (n = 14)	IgG4-RD without thyroiditis (n = 42)	*P* value
HgB (g/L)	138 ± 9	135 ± 14	0.293
WBC (10^9^/L)	6.77 ± 2.28	6.64 ± 1.59	0.843
PLT (10^9^/L)	265 ± 55	215 ± 66	0.013*
Eos% (%)	3.8 ± 6.0	4.8 ± 5.6	0.564
ESR (mm/h), M (Q1–Q3)	27 (14–36)	31 (7–56)	0.532
hsCRP (mg/L), M (Q1–Q3)	2.06 (0.33–3.99)	5.38 (0.41–5.08)	0.309
IgG (g/L)	23.56 ± 8.14	23.17 ± 14.22	0.924
IgA (g/L)	2.52 ± 1.01	2.18 ± 1.53	0.495
IgM (g/L)	1.16 ± 0.51	0.99 ± 1.09	0.616
IgG1 (mg/L), M (Q1–Q3)	12,414 (7730–15,300)	10,204 (7745–11,100)	0.149
IgG2 (mg/L), M (Q1–Q3)	6497 (3850–7870)	7039 (4235–8020)	0.782
IgG3 (mg/L), M (Q1–Q3)	413 (139–573)	732 (314–1105)	0.050*
IgG4 (mg/L), M (Q1–Q3)	4750 (2503–8505)	18,765 (2055–25,275)	0.004*
T-IgE (KU/L), M ( Q1–Q3)	100.3 (16.4–175.0)	474.3 (60.4–564.3)	0.006*
Decline of C3 (n, %)	1 (7.1)	6 (14.3)	0.666
Decline of C4 (n, %)	1 (7.1)	7 (16.7)	0.664

^*^Represented statistical significance

Eleven patients had thyroid enlargement at disease onset, and three patients had thyroid enlargement during disease progression. Comparisons of onset symptoms between IgG4-RD patients with and without thyroiditis are shown in Additional file 1: Table S1.

### Comparison with non-IgG4-related thyroiditis of HT

In 2010, Li [16] divided HT patients into two groups on the basis of IgG4 and the IgG4/IgG ratio detected by immunohistological staining: those with IgG4 thyroiditis (19 patients) and those with non-IgG4 thyroiditis (51 patients). The results demonstrated that there is a subtype of HT with unique clinical characteristics, namely, IgG4 thyroiditis. In 2019, Li [9] further explored the pathological standard of IgG4 thyroiditis by comparing it to IgG4/non-IgG4 thyroiditis of HT. To explore the difference between IgG4-related thyroiditis and classic HT, we compared IgG4-related thyroiditis patients in our study with non-IgG4-related thyroiditis of HT patients in previous studies [9, 16]. Our results revealed male predominance in IgG4-related thyroiditis patients and a higher percentage of patients with hypothyroidism than those with classic HT (*P* = 0.036, *P* < 0.001, respectively; Table 4).

**Table 4:** Comparison of IgG4-related thyroiditis in this study with Non-IgG4 related thyroiditis of HT in previous study

Demographic features	Our study IgG4-related thyroiditis	Li’s study [16] Non-IgG4 thyroiditis in HT	Li’s study [9] Non-IgG4 thyroiditis in HT	*P* value
Sample size	14	51	93	NA
Age (years)	42.8 ± 14.9	57.7 ± 8.6	58.12 ± 10.05	NA
Disease duration(yr)	4.1 ± 4.0	17.11 ± 10.34	14.88 ± 10.13	NA
Male/Female ratio	1:1	1:16	5:88	0.036*
Tg-Ab (Normal upper limit multiple)	27.58 ± 13.18	3.53 ± 6.79	1.73 (0.92–3.24)	NA
TPO-Ab (Normal upper limit multiple)	13.97 ± 4.78	2.55 ± 7.42	0.94 (0.60–1. 46)	NA
Thyroid functional status (subclinical hypo-/eu-/subclinical hyper-)	13/1/0	5/36/8	10/61/12	< 0.001*

HT: Hashimoto’s thyroiditis; Tg-Ab: serum thyroglobulin autoantibodies, TPO-Ab: thyroid peroxidase antibodies; subclinical hypo: subclinical hypothyroidism; eu: euthyroid; subclinical hyper: subclinical hyperthyroidism

*Represented statistical significance

### Treatment efficacy for IgG4-related thyroiditis

The individual clinical manifestations, treatment strategy and response are listed in Table 5. Of the 14 patients with thyroiditis, 5 (35.7%) were treated with a moderate dose of glucocorticoid (GC) monotherapy, 2 (14.3%) were treated with GCs combined with an immunosuppressant (IM) agent (GCs plus IM), 1 (7.1%) was treated with GCs combined with both an IM agent and tamoxifen (GCs plus IM and TMX), and 3 (21.4%) were treated with iguratimod. Thirteen (92.9%) patients were given levothyroxine as a supplemental treatment.

**Table 5:** Clinical features of 14 IgG4-related thyroiditis

Sex/age	Disease duration (m)	Symptoms	Affected organs	Diagnosis	Serum IgG4 (mg/L)	Thyroid US	Baseline RI	Treatment
F/52	108	Goiter, hardened thyroid, lymph node swelling, abdominal pain, arthralgia	LN, thyroid	Possible	7100	Diffuse thyroid enlargement	4	GCs 30 mg qd LS
F/56	120	Goiter, hardened thyroid, lower limb edema, chills	Kidney, pituitary, sinus, thyroid	Definite	5300	Diffuse thyroid enlargement	10	GCs 40 mg qd MMF 0.75 g bid LS
M/26	3	Goiter, hardened thyroid, neck compression	Pancreas, thyroid	Definite	11,400	Diffuse thyroid enlargement	4	GCs 50 mg qd LS
F/54	120	Goiter, hardened thyroid, neck compression, lymph node swelling	LN, thyroid	Definite	2600	Diffuse thyroid enlargement	4	GCs 40 mg qd LS
M/58	6	Goiter, hardened thyroid, neck compression	Thyroid, liver, LN	Definite	3130	Diffuse thyroid enlargement	6	GCs 40 mg qd LS
F/52	7	Goiter, hardened thyroid, neck compression, dyspnea	Thyroid	Possible	2210	Diffuse thyroid enlargement with nodular changes of the left lobe	4	Iguratimod 25 mg bid
M/31	36	Goiter, hardened thyroid	Thyroid	Definite	1850	Goiter with low-level echo in the middle of the left lobe	2	LS
M/36	5	Goiter, hardened thyroid, fatigue	Thyroid	Possible	3250	Diffuse thyroid enlargement	2	GCs 40 mg qd LS
F/16	48	Goiter, hardened thyroid	Thyroid	Possible	5130	Diffuse thyroid enlargement	2	LS
F/59	24	Goiter, hardened thyroid, neck compression, lower limb edema	Thyroid	Definite	4370	Diffuse thyroid enlargement, calcification within the right lobe	2	Iguratimod 25 mg bid LS
M/29	96	Goiter, hardened thyroid	Thyroid	Possible	16,000	Diffuse thyroid enlargement	2	Iguratimod 25 mg bid
M/61	96	Goiter, hardened thyroid, LG enlargement	SMG, LG, lung, LN, thyroid	Possible	20,600	Diffuse thyroid enlargement with solid nodules within the left lobe	10	GCs 30 mg qd CTX 100 mg qd LS
M/41	8	Goiter, hardened thyroid, nausea and vomiting	PAO, pachymeningitis, sinus, thyroid	Possible	1548	Diffuse thyroid enlargement	10	GCs 60 mg qd CTX 100 mg qd TMX 10 mg bid LS
F/28	6	Goiter, hardened thyroid	Thyroid	Possible	7540	Diffuse thyroid enlargement Multiple flaky hypoechoic areas	2	LS

Normal range of serum IgG4: 80-1400 mg/L

US: ultrasound; Baseline RI represented baseline IgG4-RD responder index; LN: lymph nodes; SMG: submandibular gland; LG: lacrimal gland; PAO: periaortitis; GCs: glucocorticoids. CTX: Cyclophosphamide. MMF: Mycophenolate Mofetil. TMX: tamoxifen. LS: levothyroxin sodium

Twelve patients were followed up for more than 6 months. At month 6, 11 (91.6%) patients had alleviation of neck compression, with the thyroid glands becoming softer according to the physical examination. The mean IgG4-RD RI decreased from 5.2 ± 3.6 to 1.6 ± 0.9 (*P* < 0.001), and the mean PGA decreased from 4.4 ± 2.4 to 2.1 ± 1.0 (*P* < 0.001). The mean serum IgG levels decreased from 20.50 ± 7.38 g/L to 14.63 ± 7.32 mg/L (*P* < 0.001), the mean serum IgG4 levels decreased from 6530 (2308–10,325) mg/L to 2924 (551–5810) mg/L (*P* = 0.004), and the mean ESR decreased from 27 (14–36) mm/h to 16 (7–14) mm/h (*P* = 0.017). There were no significant changes in CRP, Tg-Ag or TPO-Ag after treatment (*P* = 1.000, *P* = 0.841, and *P* = 0.359, respectively).

Nine patients were followed up for more than 12 months. At month 12, all patients still needed levothyroxine as a supplemental treatment, and none of them achieved a normal thyroid gland size. During the follow-up, patients still had thyroid gland enlargement confirmed by ultrasonography after 23.3 ± 17.3 months of treatment. The mean volumes of the gland before treatment and at the last follow-up were 85.10 ± 39.81 cm^3^ and 61.10 ± 40.38 cm^3^ (*P* = 0.911) for the right lobe and 55.04 ± 24.31 cm^3^ and 38.21 ± 26.11 cm^3^ (*P* = 0.777) for the left lobe, respectively. One patient’s thyroid gland returned to a normal size after 15 months of treatment.

## Discussion

IgG4-related sclerosing thyroiditis is part of the IgG4-RD spectrum, with a 2.0% prevalence in our cohort. Common symptoms of IgG4-related sclerosing thyroiditis were goiter, hard thyroid and neck compression. Most patients had irreversible hypothyroidism and needed long-term thyroxine replacement therapy. After treatment with GCs, symptoms recovered faster than changes in thyroid size on ultrasound. In addition, patients with IgG4-related thyroiditis were younger at disease onset, had fewer organs involved, a lower IgG4-RD RI and PGA and a lower frequency of submandibular gland, lacrimal gland and pancreas involvement, but they more single-organ involvement than those without thyroiditis.

IgG4-related thyroiditis was initially thought to include a wide spectrum of thyroid diseases, such as HT and RT. In 2009, based upon the immunostaining pattern of IgG4 on surgically removed thyroid specimens, Li et al. demonstrated a male predominance in patients with IgG4 thyroiditis, younger age at disease onset, elevated IgG4 concentrations, high levels of thyroid autoantibodies, diffuse low sonographic echogenicity of the thyroid and rapid development of subclinical hypothyroidism [16]. Consistent with literature reports, our study also revealed a lower female to male ratio and a higher percentage of hypothyroidism in patients with IgG4-related thyroiditis [9, 16, 21]. In HT patients, the percentage of those with subclinical hypothyroidism was approximately 50%, and less than 10% of HT patients have mild subclinical hyperthyroidism [22]. However, almost all patients with IgG4-related thyroiditis in our cohort had hypothyroidism and significantly higher levels of circulating thyroid antibodies than those with non-IgG4 thyroiditis of HT [9]. In contrast to patients with classic HT [22], patients with IgG4-related thyroiditis can have multiple organ involvement, such as the submandibular glands, lacrimal glands, and pancreas [23]. Although the underlying aetiology of IgG4+ plasma cell-rich inflammation in the thyroid remains unclear, Inomata et al. reported that the major autoantigen recognized by serum IgG4 antibodies in patients with IgG4 thyroiditis was thyroglobulin and its isoforms [22]. In a previous study, ultrasound examinations revealed that IgG4 thyroiditis was significantly correlated with diffuse hypoechogenicity, whereas non-IgG4 thyroiditis of HT was associated with diffuse coarse echogenicity [16]. Our research confirmed this finding.

It has been proposed that IgG4-RD might be divided into two distinct but overlapping subclasses according to the clinicopathological characteristics of patients: proliferative and fibrotic [4]. The separation of these subsets is grounded on disease phenotype and responsiveness to therapy. In RT, the thyroid gland may be classified into the fibrotic type, of which single-organ involvement is common [24]. Patients with the fibrotic type are more likely to have normal or mildly elevated serum IgG1, IgG4 and IgE concentrations and less hypocomplementemia and eosinophilia than patients with the proliferative type [4]. In our study, compared with patients with IgG4-RD of the proliferative subtype, patients with IgG4-related thyroiditis were younger, had lower levels of serum IgG4 and T-IgE and more single-organ involvement [25]. The organ distribution, laboratory characteristics and treatment efficacy of IgG4-related thyroiditis more likely resemble those of IgG4-RD of the fibrotic type.

At present, there is no standard treatment for IgG4-related thyroiditis. GCs are the first choice, with levothyroxine supplementation for hypothyroidism and surgical excision of the thyroid for compression [9, 26–28]. In patients who fail to respond to GCs, tamoxifen can be used as monotherapy or as add-on therapy to GCs. It can also be used initially along with GCs to reduce the risk of GC toxicity [23, 29]. Iguratimod was found to be effective when combined with GCs in the treatment of mild IgG4-RD [30] or relapsed or refractory IgG4-RD patients inadequately responding to corticosteroid treatment with or without another IM [31]. In addition, rituximab was reported to be successfully used in a patient with RT of the fibrotic type who was resistant to treatment with prednisone and tamoxifen [32]. Our study confirmed that after a long period of medical treatment, self-reported symptoms were significantly relieved, along with decreases in serum IgG levels, IgG4 levels and the ESR. However, the levels of circulating thyroid antibodies did not decrease, and the thyroid size did not change significantly after treatment with GCs. Compared with medical therapy, total thyroidectomy improves health-related quality of life and fatigue in patients with classic HT who still have symptoms despite having normal thyroid function, along with a concomitant elimination of serum anti-TPO antibodies [33]. Similarly, total thyroidectomy can quickly relieve the symptoms of IgG4-related thyroiditis, and the dosage of GCs can be reduced [26]. Additionally, the long-term use of GCs can cause a variety of adverse reactions. Therefore, for IgG4-related thyroiditis patients with single-organ involvement, if symptoms do not improve considerably after drug therapy, surgical treatment can be considered to avoid long-term adverse reactions to immunotherapy.

It is worth noting that although most patients are treated with GCs and/or IMs, long-term levothyroxine replacement is still needed due to the irreversible impairment of thyroid function. In an early pathological study of IgG4-related thyroiditis, the IgG4 thyroiditis group demonstrated thyroid-specific histological features, including the presence of small thyroid follicles, marked follicular cell degeneration, and increased giant cell/histiocyte infiltration. The severity of the IgG4 thyroiditis group was much worse than that of the non-IgG4 thyroiditis group [34] and may have been the cause of poor reversal of thyroid function. Studies have shown that hypoechogenicity of the thyroid indicates severe follicular degeneration [35]. Similarly, more diffuse hypoechogenicity on ultrasound is related to severe degeneration and the lack of normal-sized thyroid follicles in patients with IgG4 thyroiditis [34], which confirms that IgG4-related thyroiditis is associated with more severe follicle damage. Consistent with our research, the ultrasound of IgG4-related thyroiditis patients revealed decreased and uneven echogenicity, indicating a severe degree of follicle damage. Therefore, the early diagnosis and timely treatment of IgG4-related thyroiditis might be essential for minimizing irreversible organ damage or unnecessary surgical intervention.

This study had some limitations. First, the follow-up time was relatively short, and the sample size was relatively small because IgG4-related thyroiditis is rare. Second, the ultrasonography results were not confirmed by another independent researcher. Third, not all patients in this cohort underwent a thyroid biopsy.

## Conclusion

Our study indicates more single-organ involvement and lower serum IgG4 and IgE levels in IgG4-related thyroiditis than those without thyroid involvement, which is a distinct subtype of IgG4-RD and mostly resembles IgG4-RD of the fibrotic type. Goiter, hard thyroid and neck compression were the most prevalent onset symptoms observed. A low female to male ratio, the presence of circulating thyroid antibodies and a higher rate of hypothyroidism are characteristics of IgG4-related thyroiditis. Ultrasound revealed primarily asymmetric diffuse thyroid enlargement. Patient symptoms were relieved with GC therapy, but the size of the thyroid did not change significantly, and levothyroxine as a supplemental therapy was still needed.

## Supplementary Information

**Additional file 1.** Comparison of IgG4-related thyroiditis with/without thyroid enlargement as initial symptom.

## Data Availability

The corresponding author will, on request, detail the restrictions and any conditions under which access to some data may be provided.

## Abbreviations

IgG4-RD: IgG4-related disease
RT: Riedel’s thyroiditis
GD: Graves’ disease
CT: Computed tomography
MRI: Magnetic resonance imaging
PET-CT: Positron emission tomography/computed tomography
IgG4-RD RI: IgG4-RD responder index
PGA: Physician global assessment
Tg-Ab: Serum thyroglobulin autoantibodies
TPO-Ab: Thyroid peroxidase antibodies
RF: Rheumatoid factor
ESR: Erythrocyte sedimentation rate
hsCRP: Hypersensitive C-reactive protein
IgG: Immunoglobulin G
IHC: Immunohistochemical staining
CDFI: Color doppler flow imaging
PLT: Platelets
T-IgE: Total immunoglobulin E
GCs: Glucocorticoids
IM: Immunosuppressant
TMX: Tamoxifen
