# Correction to: Clinical features and treatment efficacy for IgG4-related thyroiditis

**DOI:** 10.1186/s13023-021-02071-1

**Published:** 2021-11-01

**Authors:** Xinxin Han, Panpan Zhang, Jieqiong Li, Zheng Liu, Hui Lu, Xuan Luo, Boju Pan, Xiaolan Lian, Xuejun Zeng, Wen Zhang, Xiaofeng Zeng

**Affiliations:** 1 Department of General Practice, Peking Union Medical College Hospital, Chinese Academy of Medical Science and Peking Union Medical College, State Key Laboratory of Complex Severe and Rare Diseases, Beijing, People’s Republic of China; 2 Department of Rheumatology, Peking Union Medical College Hospital, Chinese Academy of Medical Science and Peking Union Medical College, Key Laboratory of Rheumatology and Clinical Immunology, State Key Laboratory of Complex Severe and Rare Diseases, Ministry of Education and National Clinical Research Center for Dermatologic and Immunologic Diseases (NCRC-DID), No.1 Shuai Fu Yuan, Dong Cheng District, Beijing, 100730 People’s Republic of China; 3 Department of Pathology, Peking Union Medical College Hospital, Chinese Academy of Medical Science and Peking Union Medical College, State Key Laboratory of Complex Severe and Rare Diseases, Beijing, People’s Republic of China; 4 Department of Endocrine, Peking Union Medical College Hospital, Chinese Academy of Medical Science and Peking Union Medical College, State Key Laboratory of Complex Severe and Rare Diseases, Beijing, People’s Republic of China

## Correction to: Orphanet J Rare Dis (2021) 16:324 https://doi.org/10.1186/s13023-021-01942-x

It was highlighted that in the original article [1] the Funding section was incomplete. This Correction article shows the complete Funding section. The original article has been updated.
