# Supplementary material for: Clinical features and treatment efficacy for IgG4-related thyroiditis
Source: Orphanet J Rare Dis. 2021 Jul 21;16:324. doi: 10.1186/s13023-021-01942-x (PMC8293477; doi:10.1186/s13023-021-01942-x)
Supplement: Supplementary file 1 — Additional file 1. Comparison of IgG4-related thyroiditis with/without thyroid enlargement as initial symptom. [file 13023_2021_1942_MOESM1_ESM.docx]

**Supplementary Table 1: Comparison of IgG4-related thyroiditis with/without thyroid enlargement as initial symptom**

| **Parameters** | **IgG4-related thyroiditis (n=14)** | **Patients with thyroid**  **as onset symptoms**  **(n=11)** | **Patients with thyroid**  **not as onset symptoms**  **(n=3)** | ***P* value** |
| --- | --- | --- | --- | --- |
| **Demographic features** |  |  |  |  |
| Age (years) | 42.8±14.9 | 42.8±15.1 | 42.7±17.6 | 0.998 |
| Disease duration(month),  (median, min-max) | 49 (3-120) | 24 (3-120) | 96 (8-120) | 0.442 |
| Male/Female | 1:1 | 5:6 | 2:1 | 1.000 |
| Baseline IgG4-RD RI | 4.7±3.5 | 3.1±1.4 | 10.0±0 | <0.001* |
| Baseline PGA | 4.0±2.4 | 3.2±1.4 | 8.3±0.6 | <0.001* |
| Number of organs affected (median, min-max) | 2 (1-6) | 1 (1-3) | 4 (4-5) | 0.003* |
| History of allergy (n, %) | 5 (35.7) | 3 (50.0) | 2 (66.7) | 0.505 |
| **Symptoms at disease onset (n, %)** |  |  |  |  |
| lower limb edema | 2 (14.3) | 1 (9.1) | 1 (33.3) | 0.396 |
| lymph node swelling | 2 (14.3) | 2 (18.2) | 0 (0) | 0.604 |
| lacrimal gland enlargement | 1 (7.1) | 0 (0) | 1 (33.3) | 0.214 |
| abdominal pain | 1 (7.1) | 1 (9.1) | 0 (0) | 0.786 |
| arthralgia | 1 (7.1) | 1 (9.1) | 0 (0) | 0.786 |
| cough | 1 (7.1) | 1 (9.1) | 0 (0) | 0.786 |
| nausea and vomiting | 1 (7.1) | 0 (0) | 1 (33.3) | 0.214 |
| chills | 1 (7.1) | 0 (0) | 1 (33.3) | 0.214 |
| dyspnea | 1 (7.1) | 1 (9.1) | 0 (0) | 0.786 |
| fatigue | 1 (7.1) | 1 (9.1) | 0 (0) | 0.786 |
| **Organs affected (n, %)** |  |  |  |  |
| lymph node | 3 (21.4) | 3 (27.3) | 0 (0) | 0.453 |
| pancreas | 1 (7.1) | 1 (9.1) | 0 (0) | 0.786 |
| submandibular gland | 1 (7.1) | 0 (0) | 1 (33.3) | 0.214 |
| lacrimal gland | 1 (7.1) | 0 (0) | 1 (33.3) | 0.214 |
| lung | 1 (7.1) | 0 (0) | 1 (33.3) | 0.214 |
| kidney | 1 (7.1) | 0 (0) | 1 (33.3) | 0.214 |
| nasal sinus | 2(14.3) | 0 (0) | 2 (66.7) | 0.033* |
| pituitary | 2(14.3) | 0 (0) | 2 (66.7) | 0.033* |
| periaortitis/periarteritis | 1(7.1) | 0 (0) | 1 (33.3) | 0.214 |
| pachymeningitis | 1(7.1) | 0 (0) | 1 (33.3) | 0.214 |
| liver | 1(7.1) | 1 (9.1) | 0 (0) | 0.786 |
| **Laboratory examination** |  |  |  |  |
| HgB (g/L) | 138±9 | 139±9 | 138±6 | 0.810 |
| WBC (10^9^/L) | 6.77±2.28 | 6.41±2.03 | 8.10±3.14 | 0.273 |
| PLT (10^9^/L) | 265±55 | 264±55 | 270±66 | 0.885 |
| Eos% (%) | 3.8±6.0 | 2.5±4.4 | 8.6±9.7 | 0.120 |
| ESR (mm/h), (median, min-max) | 27 (7-63) | 23 (7-63) | 36 (34-38) | 0.255 |
| hsCRP (mg/L), (median, min-max) | 2.06 (0.11-4.91) | 0.58 (0.11-4.18) | 3.99 (1.57-4.91) | 0.114 |
| IgG (g/L) | 23.56±8.14 | 24.66±8.01 | 19.92±9.14 | 0.400 |
| IgA (g/L) | 2.52±1.01 | 2.40±0.98 | 2.83±1.26 | 0.558 |
| IgM (g/L) | 1.16±0.51 | 1.34±0.47 | 0.67±0.26 | 0.046* |
| IgG1 (mg/L), (median, min-max) | 12414 (5610-23800) | 12250 (7610-23800) | 11400 (5610-15300) | 0.537 |
| IgG2 (mg/L), (median, min-max) | 6497 (2220-13500) | 6395 (3400-13500) | 5190 (2220-6600) | 0.273 |
| IgG3 (mg/L), (median, min-max) | 413 (123-1530) | 290 (123-1530) | 287 (234-364) | 0.583 |
| IgG4 (mg/L), (median, min-max) | 4750 (1548-20600) | 4370 (1850-16000) | 5300 (1548-20600) | 0.400 |
| T-IgE (KU/L), (median, min-max) | 100.3 (2.0-316.0) | 59.9 (5.5-175.0) | 266.0 (2.0-316.0) | 0.068 |
| Decline of C3 (n, %) | 1 (7.1) | 0 (0) | 1 (33.3) | 0.214 |
| Decline of C4 (n, %) | 1 (7.1) | 0 (0) | 1 (33.3) | 0.214 |
